# Proteomic profiling data of HEK293 proteins bound to human recombinant renalases-1 and -2

**DOI:** 10.1016/j.dib.2018.10.137

**Published:** 2018-10-30

**Authors:** Valerii I. Fedchenko, Arthur T. Kopylov, Olga A. Buneeva, Alexei A. Kaloshin, Victor G. Zgoda, Alexei E. Medvedev

**Affiliations:** Institute of Biomedical Chemistry, 10 Pogodinskaya street, Moscow 119121, Russia

**Keywords:** Renalase, Renalase-binding protein, RP-220 peptide, Affinity-based proteomic profiling, LC–MS

## Abstract

Renalase (RNLS) is a recently discovered protein involved in blood pressure regulation. It exists both as an intracellular catalytically active flavoprotein (EC 1.6.3.5 dihydro-NAD(P):oxygen oxidoreductase) and an extracellular protein that demonstrates various cell protecting effects. Using a twenty-membered peptide corresponding to the residues 220–239 of the renalase sequence (RP-220) and the HK-2 cell line Wang et al. identified a renalase-binding protein, which was considered as a receptor for extracellular renalase crucial for MAPK signaling (Wang et al., 2015) [1]. In this study we have investigated profiles of renalase binding proteins in HEK293 cells by using affinity based proteomic profiling with full-length recombinant human RNLS-1 and human RNLS-2 as affinity ligands followed by analysis of bound proteins by liquid chromatography-mass spectrometry. Both renalases (RNLS-1 and RNLS-2) contain the RP-220 sequence (residues 220–239) but differ in their C-terminal region (residues 293–342 and 293–325, respectively). Profiling of HEK293 proteins resulted in identification of two different sets of proteins specifically bound to RNLS-1 and RNLS-2, respectively. We thus demonstrate that the C-terminal region is crucial for specific binding of renalase to its targets and/or receptors.

**Specifications table**

**Table:** 

Subject area	*Biology*
More specific subject area	*Biochemistry, Proteomics, Ligand receptor interactions*
Type of data	Analyzed LC–MS data
How data was acquired	*Affinity-based proteomic profiling of HEK-293 cell lysate by means of human recombinant renalase-1 and renalase-2 as affinity ligands followed by LC–MS/MS identification of bound proteins*
Data format	*Analyzed data in.xlsx format*
Experimental factors	*Human recombinant renalase-1 and renalase-2 were expressed in E. coli cells*
Experimental features	*HEK-293 cell lysate was loaded onto affinity sorbents (human renalases immobilized on Sepharose 4B), bound proteins were analyzed by LC–MS/MS*
Data source location	*Moscow, Russia*
Data accessibility	*Data are available as*Supplementary materials*to this paper*
Related research article	*V. Fedchenko, A. Kopylov, N. Kozlova, O. Buneeva, A. Kaloshin, V. Zgoda, A. Medvedev, Renalase secreted by human kidney НЕК293Т cells lacks its N-terminal peptide: implications for putative mechanisms of renalase action. Kidney Blood Pressure Research, 41, (2016), 593–603*

**Value of the data**

• The data can be used for discrimination of biological activity of various protein isoforms and/or their fragments obtained either during in vitro synthesis or via proteolytic processing in vivo.
• Affinity-based proteomic profiling of cell proteins specifically bound to a particular affinity ligand is a useful step in identification of potential targets (receptors) of this ligand.
• Using full length isoforms of a protein of interest and some of synthetic peptides corresponding to its fragments it is possible to determine peptide fragments crucial for interaction with cell targets.
• The lack of similarity between proteomic profiles obtained using certain peptides fragments of the entire protein (and/or its isoforms) suggest that the peptide fragments originated from the whole proteins become specific molecules unrelated to the whole parent protein
• Since profiles of HEK293 cell proteins specifically bound to human recombinant renalase-1 and human recombinant renalase-2 as affinity ligands are very specific and do not overlap, the biological activity of the RP-220 peptide identical to the renalase sequence covering residues 220–239 does not represent an intrinsic property of the renalase protein and is a characteristic feature of the 20-mer peptide.

## 1 Data

**Fig. 1:**
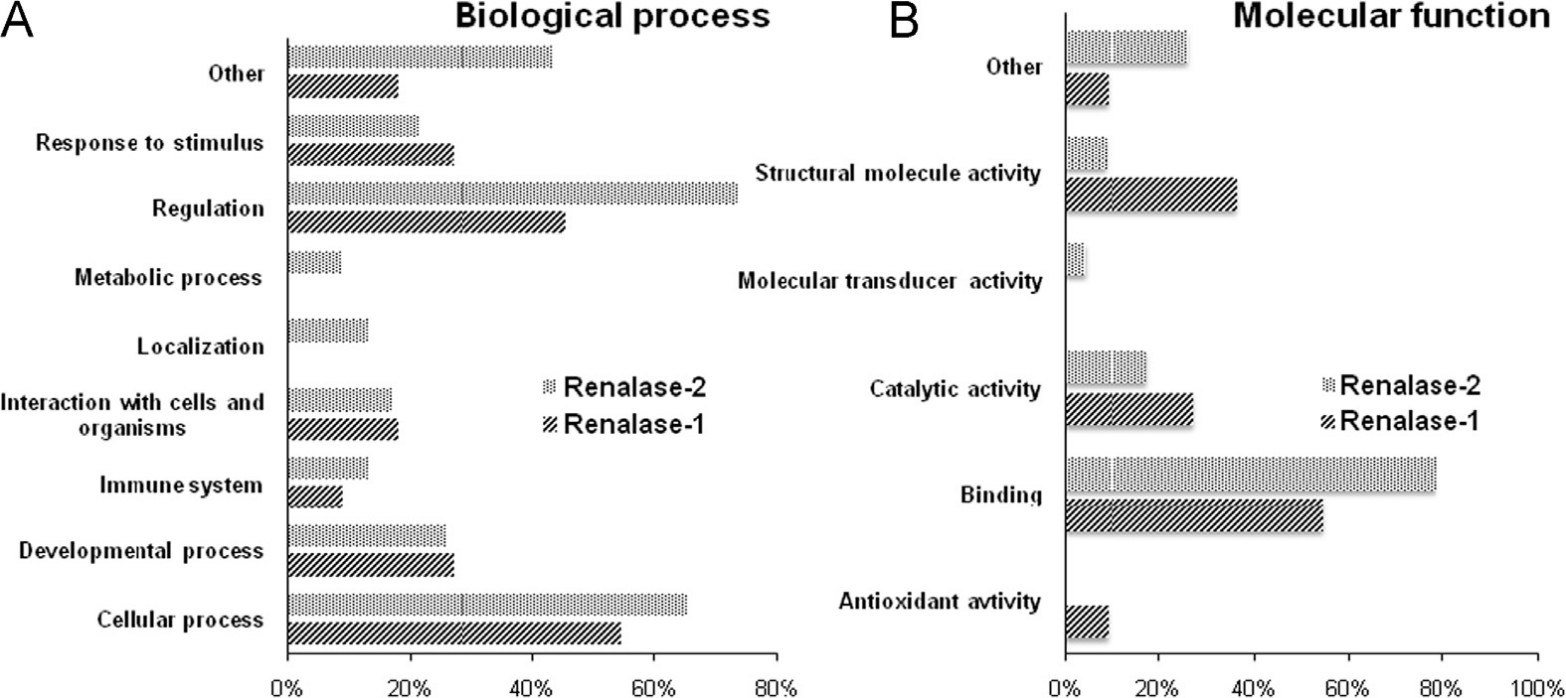
Relative contribution (in percents) of HEK293 cell proteins specifically bound to renalase-1 and renalase-2 immobilized on Sepharose 4B in Gene Ontology terms.

Proteomic profiling of HEK293 cell lysates resulted in confident identification of 11 and 23 proteins, specifically bound to renalase-1 and renalase-2 immobilized on Sepharose 4B, respectively (Supplementary file). The identified proteins bound to each affinity sorbent did not overlap. Moreover, their characteristics in Gene Ontology terms also demonstrated significant differences thus indicating a crucial role of the C-terminal region of renalases in their interaction with potential targets/receptors (Table 1and Fig. 1).

**Table 1:** Characteristics of identified HEK293 cell proteins specifically bound to renalase-1 and renalase-2 immobilized on Sepharose 4B according to Gene Ontology categories.

GO category	GO characteristics	Renalase-1	Renalase-2
**Molecular function**	Antioxidant activity	1	0
	Binding	6	18
	Catalytic activity	3	4
	Molecular transducer activity	0	1
	Structural molecule activity	4	2
	Other	1	6
**Biological process**	Cellular process	6	15
	Developmental process	3	6
	Immune system	1	3
	Interaction with cells and organisms	2	4
	Localization	0	3
	Metabolic process	0	2
	Regulation	5	17
	Response to stimulus	3	5
	Other	2	10

## 2 Experimental design, materials, and methods

### 2.1 Rationale of the study

**Fig. 2:**
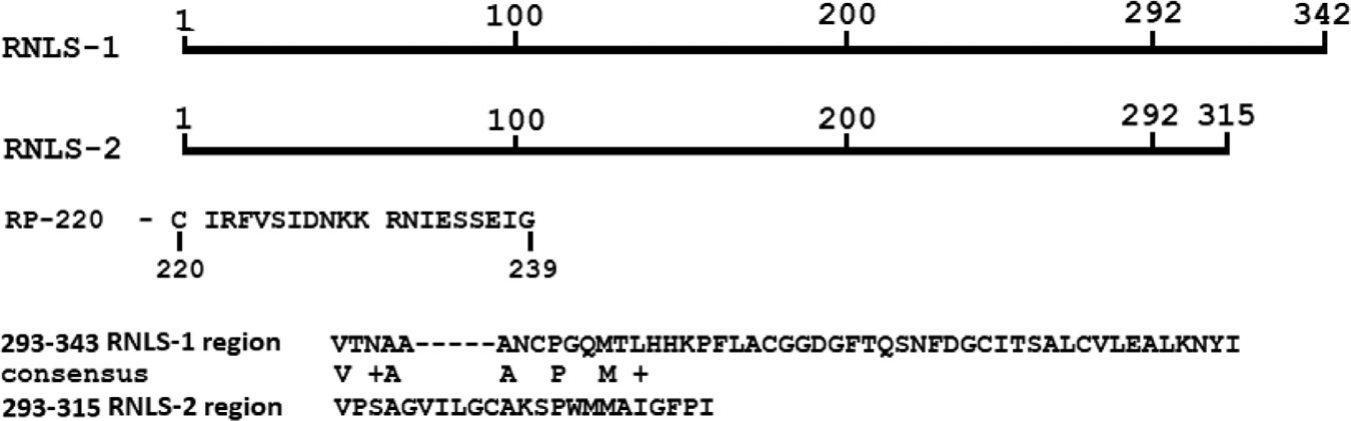
Amino acid sequences of human RNLS-1 and RNLS-2. RNLS-1 and RNLS-2 consist of 342 residues and 315 residues, respectively. Both RNLS-1 and RNLS-2 contain the RP-220 peptide (residues 220–239) but differ in the C-terminal region.

Renalase-1 and renalase-2 contain the RP-200 peptide sequence (Fig. 2) and differ by the C-terminal sequence (Fig. 2). If the RP-200 peptide sequence is actually responsible for renalase interaction with cell receptors [1] we can expect that profiles of cell proteins bound to renalase-1 and renalase-2 share similarity.

### 2.2 Expression of human recombinant renalases in *Escherichia coli* and protein purification

Expression vectors of the recombinant genes encoding renalase-1 (pET-hRen-I) and renalase-2 (pET-hRen-II) were obtained by their insertion into the into the pET-28a(+) vector by *Nco*I and *Xho*I restriction sites [2]. The resultant expression vectors pET-hRen-I and pET-hRenII were then transformed into *E. coli* Rosetta (DE3) cells [2]. Transformation was performed using the conventional method [3]. Both renalases expressed as proteins, containing the C-terminal hexahistidine tag, were purified by affinity chromatography on Ni-Sepharose.

### 2.3 Preparation of affinity sorbents containing human renalase-1 and -2 and isolation of renalase-binding HEK293 proteins

Human recombinant renalases-1 and -2 were immobilized on cyanogen bromide-activated-Sepharose 4B by means of a conventional protocol [4]. For evaluation of nonspecific binding control cyanogen bromide-activated-Sepharose 4B was subjected to the same treatments as the renalase-Sepharose but without immobilized renalase-1 or renalase-2.

Eukaryotic HEK293T cells cultured in 10 cm-Petri dishes in DMEM medium containing 10% FCS, 4 mM glutamine, and antibiotics penicillin and streptomycin (50 units/ml and 50 μg/ml, respectively) were scraped off from the substrate, washed with saline, and harvested by centrifugation [5]. After homogenization in 50 mM phosphate buffer, pH 7.4, using the Heidolph MR 100 homogenizer cells were lyzed with 3% X-100 for 35 min at 4 °C. The supernatant obtained after centrifugation at 16,000 rpm (Eppendorf 5415 R centrifuge) for 5 min was used for affinity based proteomic profiling.

The cell supernatant was loaded onto the affinity sorbent in 50 mM potassium phosphate buffer, pH 7.4, containing 0.3 M NaCl, and incubated overnight in suspension (1:1) under gentle stirring at 4 °C. After washing with the same buffer up to protein disappearance controlled spectrophotometrically (OD_280_), the sorbent with affinity-bound proteins was packed into a column (0.5 cm × 1 cm) and the proteins bound to the affinity ligands (RNLS-1 or RNLS-2) were eluted using 0.1 M glycine buffer (pH 2.8) containing 3 M NaCl (at a flow rate 0.5 ml/min). The eluate was concentrated using an Amicon Ultra (10 kDa) centrifugal filter unit and precipitated with the help of chloroform-ethanol mixture [6].

### 2.4 LC–MS parameters

Mass spectrometry analysis was performed on an Orbitrap Fusion (Thermo Scientific, USA) with installed ESI-NSI ion source. The instrument was operated in positive ionization mode and voltage was set at 2.1 kV and drying gas temperature at 250 °C. Precursor ions with charge states from *z* = 2+ to *z* = 6+ were surveyed within a range of 400–1200 *m/z* with AGC (acquisition gain control) of 2e5 ions, or maximum integration time of 35 ms. Ions were isolated using a quadrupole mass analyzer within ±1.5 *m*/*z* (offset 0.5 *m*/*z*) and passed to fragmentation in HCD (high-energy collision dissociation) mode following acquisition in an ultra-high field orbital detector. The acquisition was controlled under “top speed” mode for 3 s and fragmentation spectra were recorded in a range from 110 *m/z* to 2100 *m/z* with AGC set to 4e5 ions, or maximum integration time of 85 ms.

Chromatography separation was carried out on an Ultimate 3000 RSLCnano (Thermo Scientific, USA). Samples were loaded onto an enrichment Acclaim µ-Precolumn (0.5 mm × 3 mm, 5 µm) (Thermo Scientific, USA) at a flow rate 15 µL/min for 3.5 min in 2% acetonitrile supplied by 0.1% formic acid and 0.03% acetic acid. Analytical separation was carried out at a flow rate 0.3 µL/min using an Acclaim Pepmap® C18 (75 µm × 150 mm, 2 µm) (Thermo Scientific, USA) column in a gradient of mobile phase A (water with 0.1% formic acid and 0.03% acetic acid) and mobile phase B (acetonitrile with 0.1% formic acid and 0.03% acetic acid) in the following gradient: 2–37% of mobile phase B for 45 min following column washing in 90% of mobile phase B for 8 min and equilibrating the column in initial gradient conditions (2% of mobile phase B) for 15 min before starting the next run.

### 2.5 Mass spectrometry data treatment

Raw data files were converted in MGF-files using MSConvert (Proteowizard). Peak lists obtained from converted spectra were identified using X!Tandem version X!Tandem Vengeance (2015.12.15.2). The search was conducted using SearchGUI version 3.3.0 [7]. Protein identification was conducted against a concatenated target/decoy version of the *Homo sapiens* complement of the UniProt fasta (release February, 2018). The decoy sequences were created by reversing the target sequences in SearchGUI. The identification settings were as follows: trypsin (specific), with a maximum of 1 missed cleavage within ±5.0 ppm tolerance as MS1 level and ±0.01 Da tolerance for MS2 tolerances. The following variable modifications were set: carbamidomethylation of C (+57.021464 Da) and oxidation of M (+15.994915 Da) and deamidation of N and Q (+0.98402 Da). Variable modifications refined after search procedure. Peptides and proteins were inferred from the spectrum identification results using PeptideShaker version 1.16. Peptide Spectrum Matches (PSMs), peptides and proteins were validated at a 1.0% False Discovery Rate (FDR) estimated using the decoy hit distribution.

The lists of identified HEK293 proteins bound to the affinity sorbents (renalase-1-Sepharose and renalalase-2-Sepharose) were corrected for nonspecific binding to the control (renalase-free) Sepharose sorbent. The list of nonspecifically bound proteins is given separately (See Supplementary file).
