# Supplementary material for: Proteomic profiling data of HEK293 proteins bound to human recombinant renalases-1 and -2
Source: Data Brief. 2018 Oct 30;21:1477–82. doi: 10.1016/j.dib.2018.10.137 (PMC6234383; doi:10.1016/j.dib.2018.10.137)
Supplement: Supplementary file 1 — Supplementary material [file mmc1.docx]

All the authors of the manuscript entitled  **Proteomic profiling data of HEK293 proteins bound to human recombinant renalases-1 and -2**

Valerii I. Fedchenko, Arthur T. Kopylov, Olga A. Buneeva, Alexei A. Kaloshin, Victor G. Zgoda, Alexei E. Medvedev

DECLARE THAT THEY HAVE NO CONFLICT OF INTEREST.

On behalf of all coauthors


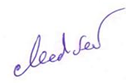


Professor Alexei Medvedev, MD, PhD, DSci
